# Activation of NO-cGMP Signaling Rescues Age-Related Memory Impairment in Crickets

**DOI:** 10.3389/fnbeh.2016.00166

**Published:** 2016-08-26

**Authors:** Yukihisa Matsumoto, Chihiro S. Matsumoto, Toshihumi Takahashi, Makoto Mizunami

**Affiliations:** ^1^College of Liberal Arts and Science, Tokyo Medical and Dental UniversityIchikawa, Japan; ^2^Graduate School of Life Science, Hokkaido UniversitySapporo, Japan; ^3^Graduate School of Life Sciences, Tohoku UniversitySendai, Japan

**Keywords:** aging, nitric oxide, olfactory learning, long-term memory, cricket

## Abstract

Age-related memory impairment (AMI) is a common feature and a debilitating phenotype of brain aging in many animals. However, the molecular mechanisms underlying AMI are still largely unknown. The cricket *Gryllus bimaculatus* is a useful experimental animal for studying age-related changes in learning and memory capability; because the cricket has relatively short life-cycle and a high capability of olfactory learning and memory. Moreover, the molecular mechanisms underlying memory formation in crickets have been examined in detail. In the present study, we trained male crickets of different ages by multiple-trial olfactory conditioning to determine whether AMI occurs in crickets. Crickets 3 weeks after the final molt (3-week-old crickets) exhibited levels of retention similar to those of 1-week-old crickets at 30 min or 2 h after training; however they showed significantly decreased levels of 1-day retention, indicating AMI in long-term memory (LTM) but not in anesthesia-resistant memory (ARM) in olfactory learning of crickets. Furthermore, 3-week-old crickets injected with a nitric oxide (NO) donor, a cyclic GMP (cGMP) analog or a cyclic AMP (cAMP) analog into the hemolymph before conditioning exhibited a normal level of LTM, the same level as that in 1-week-old crickets. The rescue effect by NO donor or cGMP analog injection was absent when the crickets were injected after the conditioning. For the first time, an NO donor and a cGMP analog were found to antagonize the age-related impairment of LTM formation, suggesting that deterioration of NO synthase (NOS) or molecules upstream of NOS activation is involved in brain-aging processes.

## Introduction

Impairment of memorization ability in relation to aging (Age-related memory impairment, AMI) is a common feature of brain aging in many animal species, including humans (Yankner et al., 2008), insects (Tamura et al., 2003; Mery, 2007) and nematodes (Murakami and Murakami, 2005). Recovery from AMI is one of the major challenges in human lives in order to maintain quality of life. To address this issue, it is important to clarify the neuronal and molecular mechanisms underlying AMI in the brain. However, the long life spans of rodents (age at first reproduction in mice and rats being 35–50 days and maximum longevity being 4 years (Austad, 1997)), generally used as mammalian model animals for aging studies, have always been an obstacle to study AMI.

Compared to mammals, insects have relatively short life cycles that would facilitate investigation of age-related changes. Insects have been extensively used for learning and memory studies because they have high learning abilities subtended by a simpler neural system and smaller number of neurons than those in vertebrates (Mizunami et al., 2004; Giurfa, 2007). Thus, insects are suitable for AMI studies. In insects, AMI has been reported in fruit-flies (Tamura et al., 2003; Mery, 2007), honeybees (Tofilski, 2000; Farooqui, 2007; Behrends and Scheiner, 2010) and cockroaches (Brown and Strausfeld, 2009). Only a few studies on the fruit-fly *Drosophila* have shed light on the molecular mechanisms of AMI (Tamura et al., 2003; Yamazaki et al., 2007, 2010).

In *Drosophila*, single cycle training which consists of two trials, one trial pairing an odor with electric shock and another trial presenting another odor alone, leads to formation of several successive phases of memory: short-term memory (STM), middle-term memory (MTM) and short-term or middle-term anesthesia-resistant memory (ARM; Bouzaiane et al., 2015). Tamura et al. (2003) reported that among the three memory phases after single cycle training only MTM was impaired with aging. MTM formation is known to be dependent on neuropeptides coded by *amn*, but overexpression of *amn* in dorsal-paired medial (DPM) neurons could not improve AMI (Tamura et al., 2003). This failure in rescuing MTM formation suggests the involvement of cyclic AMP (cAMP) signaling (adenylyl cyclase (AC)-cAMP-PKA pathway) degradation in addition to defect in *amn* transcripts because the cAMP pathway is presumably downstream of *amn* signaling. Another study on *Drosophila* showed AMI in long-term memory (LTM), a phase of memory that is formed after repeated multiple-trial conditioning with adequate intervals (spaced training) and is protein synthesis-dependent (Mery, 2007). However, the molecular basis underlying AMI of LTM in insects remains unknown.

Crickets (*Gryllus bimaculatas*), the experimental animals used in the present study, are easy to maintain in a laboratory environment under age control. Crickets are capable of learning olfactory signals quickly and memorizing them practically for a lifetime (Matsumoto and Mizunami, 2002a,b, 2004), and they can easily be used for detailed pharmacological studies (Matsumoto et al., 2003, 2006, 2009). In our previous studies, we investigated the biochemical basis of LTM formation by behavioral pharmacological experiments. In young adult crickets, memory formed by single-trial conditioning (pairing of an odor with reward) decline in several hours, while memory formed by two sets of differential conditioning (a set of differential conditioning consists of two trials pairing an odor with reward and another odor with punishment) with intervals (spaced training) is maintained for several days (Matsumoto et al., 2006). The latter memory formed by multiple spaced training is impaired by a protein synthesis inhibitor, and the memory retention curve of this experiment well resembles that after the single-trial conditioning. Therefore, the protein synthesis inhibitor discriminates the two distinct memory phases: protein synthesis-dependent memory and protein synthesis-independent memory (Matsumoto et al., 2003). The former type of memory is referred to as LTM. The latter type is further classified into two memory phases according to the sensitivity to anesthetic treatment: anesthesia-sensitive memory (ASM) that disappears within 20 min after conditioning, and ARM that reaches its peak from 20 min to around 3 h after conditioning (Matsumoto and Mizunami, 2002a). When a nitric oxide (NO)-cyclic GMP (cGMP) signaling pathway inhibitor or cAMP-PKA signaling pathway inhibitor was injected before the spaced training, LTM was fully impaired, while ARM remained intact (Matsumoto et al., 2006, 2009). On the other hand, injection of activators of the NO-cGMP pathway or activators of the cAMP-PKA pathway prior to single-trial conditioning induced the formation of LTM (Matsumoto et al., 2006). Thus, in young adult crickets, the NO-cGMP pathway and its downstream cAMP-PKA pathway are necessary and sufficient for LTM formation (Matsumoto et al., 2006).

In the present study, we investigated whether crickets exhibit AMI in olfactory learning and found that crickets aged 3 weeks after the final molt show AMI in LTM formation but neither in ARM formation nor in LTM retrieval. Moreover, the AMI in LTM formation was fully compensated by injecting activators of the NO-cGMP pathway or cAMP pathway. This is the first demonstration where activation of the NO-cGMP signaling pathway prevents AMI in LTM, suggesting that the deterioration of NO synthase (NOS) or molecules in upstream signaling pathways is critical in brain-aging processes.

## Materials and Methods

### Materials

Adult male crickets, *Gryllus bimaculatus*, reared in a 12-h light-dark cycle at 27 ± 2°C, were used for behavioral experiments. Because a portion of females showed oviposition behavior to the odor sources, only male crickets were used in behavioral experiments. We define crickets until 24 h after the final molt as 1-day-old crickets. Newly enclosed male and female adult crickets were removed from the plastic rearing containers (35 cm × 30 cm × 70 cm) every day and housed in another plastic container, ensuring that all adults used in the experiments were of known age. Only the crickets with intact antennae were used for the experiments. Two days before the start of the experiment, male crickets were individually placed in 100-ml glass beakers and fed a diet of insect pellets *ad libitum*, but they were deprived of drinking water to enhance their motivation to search for water. In the present work, 1-week-old crickets correspond to 4 to 7-day-old crickets at the start of the conditioning. Two-week, 3-week and 4-week-old crickets correspond to 11–14-day, 18–21-day and 25–28-day-old crickets at the start of the conditioning, respectively. All experiments were carried out in photophase.

### Classical Olfactory Conditioning

Crickets were trained by multiple trials of an olfactory differential conditioning procedure, which leads to more robust memory compared to that produced by the same number of elemental conditioning trials (Matsumoto and Mizunami, 2002a). The conditioning procedure has been described previously (Matsumoto and Mizunami, 2002a). In short, individual crickets placed in beakers were given differential conditioning trials using 1-ml hypodermic syringes. One set of differential conditioning consists of two trials, one to associate peppermint odor with water reward (appetitive conditioning) and the other to associate vanilla odor with salt water punishment (aversive conditioning). Two sets or four sets of differential conditioning trials with 5-min intervals were given to crickets. A small filter paper attached to the needle of the syringe at 10 mm from its tip was soaked with odorant solution. The syringes used for appetitive conditioning and aversive conditioning were filled with water and 20% NaCl solution, respectively. For conditioning, the filter paper was placed within 1 cm of the cricket’s head and 2 s later a drop of water or salt water was placed at the mouth of the cricket for 2 s. After the cessation of training, each cricket was given a diet of insect pellets *ad libitum* in a beaker until it was subjected to an odor preference test.

### Odor Preference Test

Odor preference tests were performed a few hours before the onset of conditioning and at 30 min, 2 h, 4 h or 1 day after the end of conditioning. Animals were placed in a test apparatus and were allowed to choose between reward-associated odor (peppermint) and punishment-associated odor (vanilla), as described previously (Matsumoto and Mizunami, 2002a). On the floor of the “test chamber” of the apparatus, there were two circular holes that connected the chamber with two of three sources of odor. Each odor source is a cylindrical plastic container containing a filter paper soaked with 3-μl solution of vanilla or peppermint essence, covered with a fine gauze-net. The three containers were mounted on a rotatable holder.

Before the preference test, each animal was transferred from the beaker to the “waiting chamber” of the apparatus and left for 4 min to become accustomed to the surroundings, and then the door to the test chamber was opened. The test started when the animal entered the test chamber and lasted for 4 min. Two minutes later, the relative positions of the vanilla and peppermint sources were changed by rotating the container holder. An odor source was considered to have been visited when the animal probed the top net with its mouth. The time spent for visiting each odor source was measured cumulatively. If the total time of visits of an animal to either source was less than 10 s we considered that the animal was less motivated to visit odor sources, possibly due to poor physical condition, and the data were rejected. At the end of the training, the sliding door was opened and the animal was gently pushed into the waiting chamber and then transferred to a beaker. After completing the test session, animals were provided with a diet of insect pellets *ad libitum*.

### Anesthetic Treatment with CO_2_

To determine the effect of anesthetic treatment at different timing, animals were anesthetized with CO_2_ at 0 min, 10 min or 20 min after one set of differential conditioning with ITI of 5 min. After treating the animals with CO_2_ for 30 s, the air in the beaker was ventilated for 10 s. Animals began to move their legs approximately 2 min after the cessation of CO_2_ treatment and recovered normal locomotion within 5 min.

### Pharmacology

Animals were injected with 3-μl cricket saline (Matsumoto et al., 2003) containing drugs into the hemolymph of the head using a 10-μl microsyringe (WPI, Tokyo, Japan). S-nitroso-n-acetyl-penicillamine (SNAP), 8-bromoguanosine 3′:5′-cyclic monophosphate (8-br-cGMP) and 8-bromoadenosine 3′:5′-cyclic monophosphate (8-br-cAMP) were purchased from SIGMA (Tokyo, Japan).

### Data Analysis

Log-rank test was used to compare life spans after the final molt between male and female crickets. Relative odor preference of each animal was measured using the preference index (PI) for rewarded odor (peppermint; %), defined as *t*_P_/(*t*_P_+*t*_V_) × 100, where *t*_P_ is the time spent exploring the peppermint source and *t*_V_ is the time spent exploring the vanilla source. Wilcoxon’s (WCX) test was used to compare odor preferences in different tests of a given animal group. The Mann-Whitney *U* (M-W) test was used to compare odor preferences of different groups. The Kruskal-Wallis (K-W) test was used to compare times spent exploring odor sources or odor preferences among three or more groups. For multiple comparisons, Holm’s method was used to adjust the significance level.

## Results

### Life Span of Crickets

**Figure 1:**
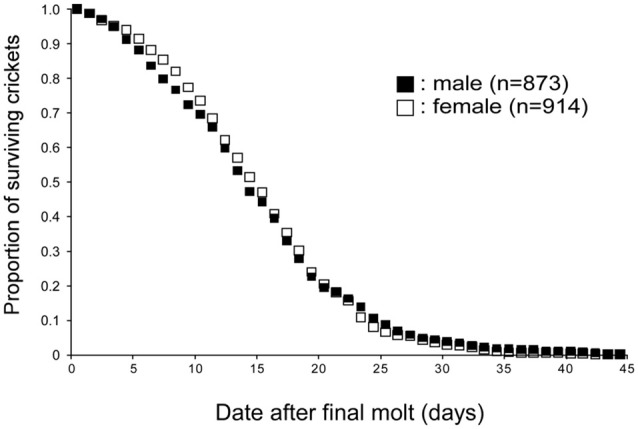
**Life spans of crickets.** Survival curves of male and female crickets, *Gryllus bimaculatus*. The data were obtained from 873 male and 914 female crickets. Average life spans of male and female crickets are both 14 days from the final molt.

For investigating the life span of adult crickets, approximately 200 to 300 newly molted crickets were isolated from the rearing container and the number of dead crickets was counted every day (Figure 1). The environment of isolation containers (e.g., size, food, water, temperature, humidity) was identical to that of the rearing container. The male to female ratio within the group was approximately 1:1. The experiment was replicated seven times and the data were pooled. In our breeding conditions, the average life spans of male and female crickets were 14.8 days (SE ± 0.3 days) and 15.1 days (SE ± 0.2 days) after the final molt, respectively. No significant difference in life span after the final molt was found between male and female crickets (*p* = 0.9871, log-rank test).

### One-day Memory Retention in Aged Adult Crickets

**Figure 2:**
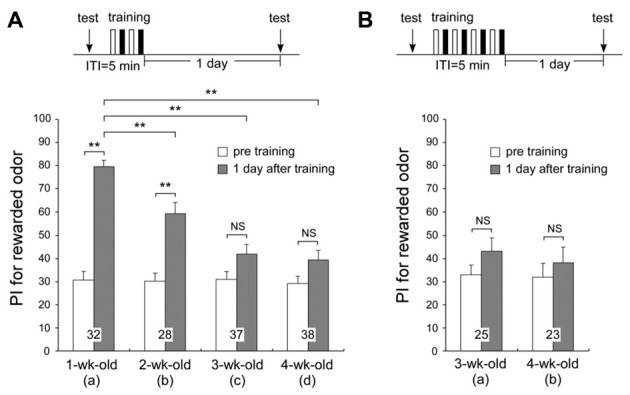
**One-day memory retention in crickets of different ages.** Four groups of different-aged crickets were subjected to two sets of differential conditioning (2-set group) **(A)**. Two groups of different-aged crickets were subjected to four sets of differential conditioning (4-set group) **(B)**. The time schedule of the experiment is shown above the figure (white bars stand for appetitive conditionings and black bars stand for aversive conditionings under “training”). Preference indexes (PIs) for rewarded odor before conditioning (white bars) and 1 day after conditioning (shaded bars) in all animals are shown as means ± SE. The results of statistical comparisons within each group (Wilcoxon’s, WCX test) or between groups (Mann-Whitney U, M–W), adjusted by Holm’s method, are shown as asterisks (**p < 0.01). The number of animals tested is shown at each data point.

We compared the times spent exploring odor sources in the test before multiple-trial conditioning among different age groups. The times spent exploring odor sources were not significantly different between any of the age groups (*t*_p_ + *t*_v_: means ± SE, 30.1 ± 3.0 s for 1-week-old, 22.1 ± 2.4 s for 2-week-old, 22.1 ± 2.0 s for 3-week-old, and 23.2 ± 1.8 s for 4-week-old crickets, *p* = 0.0655, *T* = 7.2583, *df* = 3, K-W test, shown in Figure 2A). This value is assumed to reflect the motivation level of the cricket group. Though not significant, 1-week-old crickets spent slightly more time exploring the odor sources than the older age groups, while the total exploration time values were comparable among the 2-week, 3-week, and 4-week-old groups. It suggests that although the motivation may slightly fall in the early adulthood, it does not have a critical effect on LTM formation, because 2-week-old group shows significant level of LTM while 3-week-old and 4-week-old groups do not (Figure 2A, described later). No significant difference in the innate PI for peppermint was found among the different age groups (Figure 2A, PI for peppermint: means ± SE, 30.8 ± 3.5 for 1-week-old, 30.3 ± 3.5 for 2-week-old, 31.2 ± 3.5 for 3-week-old, and 29.2 ± 3.4 for 4-week-old crickets, *p* = 0.9677, *T* = 0.2583, *df* = 3, K-W test), indicating that odor discrimination capability and innate odor preference of aged adults (3-week-old and 4-week-old crickets) are similar to that of young adults (1-week-old crickets).

To clarify whether crickets exhibit age-related impairment in olfactory memory, we examined 1-day memory retention of multiple-trial conditioning in four different age groups of adults (Figure 2). For 1-week-old crickets, memory at 1 day after multiple-trial conditioning has been shown to be LTM, which is protein synthesis-dependent (Matsumoto et al., 2003). Four groups of different-aged crickets were subjected to two sets of differential conditioning (2-set group) and tested 1 day after conditioning. The 1-week-old group exhibited significant level of LTM: preference for peppermint odor was significantly greater than that before conditioning (Figure 2Aa, *p* < 0.0001, WCX test). The 2-week-old group also exhibited significant level of LTM (Figure 2Ab, *p* < 0.0001, WCX test), but the memory retention level of the 2-week-old group was significantly less than that of the 1-week-old group (compared to Figure 2Aa, *p* = 0.0013, M-W test). Neither the 3-week-old nor the 4-week-old group exhibited a significant level of 1-day memory retention: odor preferences did not significantly differ from those before conditioning (3-week-old, Figure 2Ac, *p* = 0.0742; 4-week-old, Figure 2Ad, *p* = 0.0733, WCX test). Retention levels of the 3-week-old and the 4-week-old groups were significantly less than that of the 1-week-old group (compared to Figure 2Aa, *p* < 0.0001, M-W test). We trained 3-week-old and 4-week-old crickets with two additional sets of differential conditioning to test whether increased trials could result in the formation of LTM in aged crickets (Figure 2B). However, 3-week-old and 4-week-old adults subjected to four sets of differential conditioning (4-set group) also exhibited no significant level of 1-day retention (Figure 2Ba, *p* = 0.2087; Figure 2Bb, *p* = 0.5201, WCX test), indicating severe LTM impairment in aged crickets that cannot be restored by two-times larger number of trials than that which are sufficient for young adults. These results indicate that AMI in LTM occurs from 2 weeks after the final molt and reaches a plateau level at 3 weeks after the final molt.

### Memory Retention Curve after Conditioning in Aged Crickets

**Figure 3:**
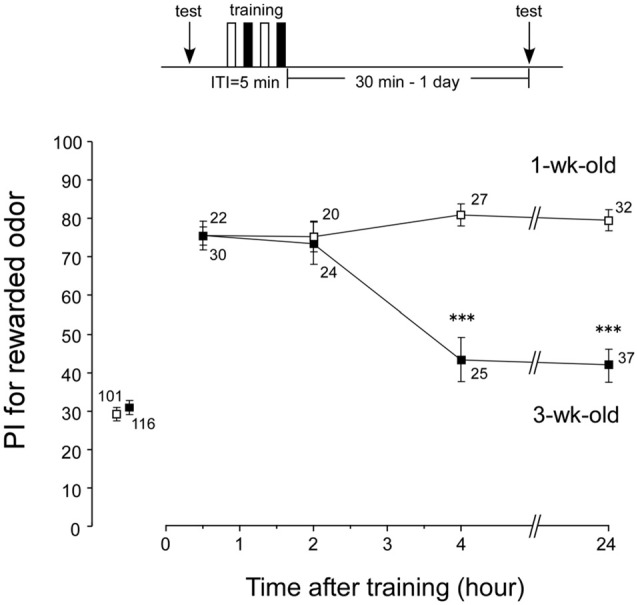
**Memory retention curves after conditioning in 1-week-old and 3-week-old crickets.** Four groups of 1-week-old crickets (open squares) and another four groups of 3-week-old crickets (black squares) were subjected to two sets of differential conditioning. Odor preference tests were given to all animals before and at various times after conditioning. The time schedule of the experiment is shown above the figure. PIs for rewarded odor are shown as means ± SE. To simplify the figure, the PIs before conditioning are shown as pooled data from four 1-week-old or four 3-week-old groups. Statistical comparisons of odor preferences were made between 1-week-old and 3-week-old groups at each time after conditioning (M-W test), and the results are shown above each data point (***p < 0.001). The number of animals is shown at each data point.

Loss of LTM in aged crickets could be due to unsuccessful conditioning. Were memories in earlier phases (e.g., ASM and ARM) acquired normally in aged crickets? We investigated memory retention curves after conditioning in both aged crickets (3-week-old crickets) and young adult crickets (1-week-old crickets). Four groups of 1-week-old crickets and four groups of 3-week-old crickets were subjected to two sets of differential conditioning. The odor preferences of animals were tested before conditioning and at 30 min, 2 h, 4 h and 24 h after conditioning. The 1-week-old group exhibited a significant level of 30-min retention (Figure 3, *p* < 0.0001, WCX test) and exhibited no significant decay of memory retention at 2 h, 4 h and 24 h (Figure 3, *p* = 0.3893, *df* = 3, K-W test). At 30 min and 2 h after conditioning, the memory retention levels of the 3-week-old groups were not significantly different from those of the 1-week-old groups (Figure 3, 30-min retention, *p* = 0.6698; 2-h retention, *p* = 0.4574, M-W test). This indicates that aged crickets have: (1) normal sensory and motor functions necessary for good learning performance; (2) normal initial acquisition of memory; and (3) normal memory retention up to 2 h after conditioning. However, the 3-week-old groups exhibited a significant decay of memory retention from 4 h after conditioning (compared to 1-week-old groups, *p* < 0.0001, M-W test), with no significant level of memory (*p* = 0.0975, WCX test). These results indicate that AMI occurs in LTM, but not in ARM, in crickets.

### Effects of Anesthetic Treatment with CO_2_ in 1-week-old and 3-week-old Crickets

**Figure 4:**
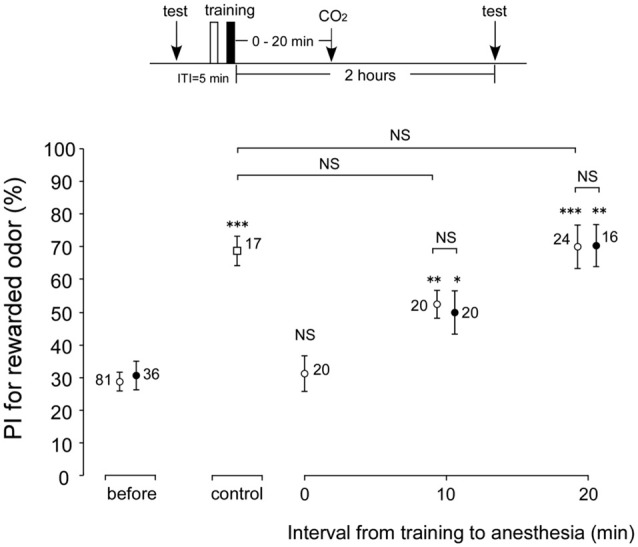
**Effects of anesthetic treatment with CO_2_ in 1-week-old and 3-week-old crickets.** Three groups of 1-week-old crickets (open circles) and two groups of 3-week-old crickets (black circles) were subjected to one set of differential conditioning with an ITI of 5 min, and then received anesthetic treatment. Animals were anesthetized with CO_2_ for 30 s either immediately (0 min) after conditioning, at 10 min or 20 min after conditioning. Another group of 1-week-old crickets (open square) was subjected to one set of differential conditioning without anesthetic treatment (control). Odor preference tests were given to all animals before and at 2 h after conditioning. The time schedule of the experiment is shown above. PIs for rewarded odor are shown as means ± SE. To simplify the figure, the PIs before conditioning are shown as pooled data from four 1-week-old or two 3-week-old groups (before). The results of statistical comparisons within each group (WCX test) or between groups (M-W test), adjusted by Holm’s methods, are shown as asterisks (*p < 0.05, **p < 0.01, ***p < 0.001, NS *p* > 0.05). The number of animals is shown at each data point.

In the experiments described above, memory retention scores at 30 min and 2 h after conditioning were not affected by AMI. However, whether these memories are actually ARM is not clear. To reveal whether cricket ARM is affected by AMI, we compared the retention scores after post-conditioning anesthetic treatment in young adult (1-week-old) and aged (3-week-old) crickets. Four groups of young adult crickets went through one set of differential conditioning, received no anesthetic treatment (control group) or 30 s of CO_2_ anesthetic treatment at 0 min or 10 min or 20 min after conditioning. All groups were tested for their memory retention at 2 h after conditioning. Compared with the retention score before conditioning, the score at 2 h after conditioning in the control group without anesthetic treatment showed significant increase (Figure 4, *p* = 0.0008, WCX test), while the 2-h retention score of the group with immediate post-conditioning anesthesia (0 min group) exhibited no significant difference (Figure 4, *p* = 0.4688, WCX test). Thus, the memory observed immediately after conditioning in the control group is classified as the ASM. In contrast, memory retention scores in young adult groups with anesthetic treatment at 10 or 20 min after conditioning increased significantly in comparison with that before conditioning (Figure 4, 10-min group, *p* = 0.0019; 20-min group, *p* = 0.0003, WCX test). The memory components observed in these groups are classified as that belonging to ARM. The 10-min young adult group showed retention score that was significantly lower than that of the control group (Figure 4, *p* = 0.0073, compared to non-anesthetized group, M-W test). The difference component between these memory retention scores is the labile ASM. Retention score of the young adult 20-min group did not significantly differ from that of the non-anesthetized control (Figure 4, *p* = 0.5505, M-W test). Therefore, it indicates that ASM disappears before 20 min. Meanwhile, the 3-week-old cricket groups that went through post-conditioning anesthesia either at 10 min or at 20 min exhibited significant increase in 2-h retention scores compared to the innate score of aged crickets (Figure 4, 10-min group, *p* = 0.0373; 20-min group, *p* = 0.0061, WCX test), indicating the existence of ARM in aged crickets. Comparisons of these ARM scores of aged crickets with those of the corresponding young adult crickets did not show significant differences (Figure 4, 10 min group, *p* = 0.8392; 20 min group, *p* = 0.6172, compared to respective 1-week-old group, M-W test). From these results, we conclude that ARM is not affected by aging.

### Effects of NO-donor and cGMP Analog on LTM in Aged Adult Crickets

**Figure 5:**
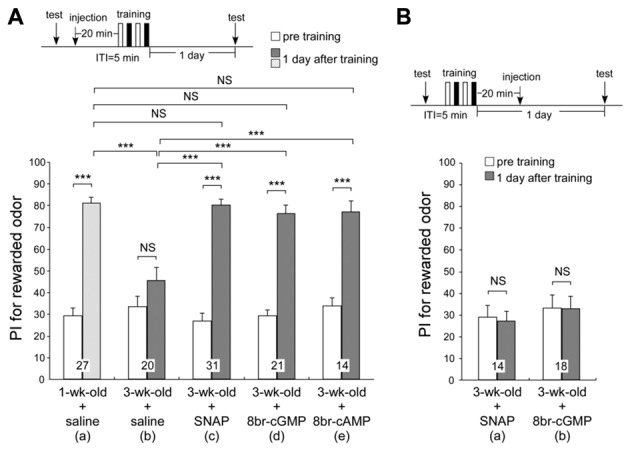
**Effects of NO-donor and cyclic GMP (cGMP) analog on long-term memory (LTM) in aged adult crickets.** One group of 1-week-old crickets and five groups of 3-week-old crickets were subjected to two sets of differential conditioning. In **(A)**, the 1-week-old cricket group was injected with 3-μl saline into the hemolymph **(a)** and three groups of 3-week-old crickets were each injected with 3-μl saline alone **(b)**, saline containing 200 μM of an NO donor, S-nitroso-n-acetyl-penicillamine (SNAP) **(c)**, or 200 μM of a cyclic GMP (cGMP) analog, 8-bromoguanosine 3′:5′-cyclic monophosphate (8br-cGMP) **(d)**, into the hemolymph at 20 min prior to conditioning. In **(B)**, two groups of 3-week-old crickets were each injected with 3-μl saline containing 200-μM SNAP **(a)** or 200-μM 8br-cGMP **(b)** into the hemolymph at 20 min after conditioning. The time schedule of the experiment is shown above the figure. PIs for rewarded odor before conditioning (white bars) and 1 day after conditioning (hatched bar in 1-week-old crickets; shaded graphs in 3-week-old crickets) are shown as means ± SE. The results of statistical comparisons within each group (WCX test) or between groups (M–W test), adjusted by Holm’s method, are shown as asterisks (***p < 0.001). The number of animals tested is shown at each data point.

In our previous work, the memory retention curve after differential conditioning in 3-week-old crickets was similar to that of young adult crickets injected with a NOS inhibitor, L-NAME, a soluble guanylyl cyclase (sGC) inhibitor, ODQ or an AC inhibitor, KT5720, into the hemolymph prior to differential conditioning (Matsumoto et al., 2006). These retention curves were also similar to those of young adult crickets trained with single-trial conditioning, which does not induce formation of LTM. We also found that injection of an NO donor, cGMP analog or cAMP analog prior to single-trial conditioning induced formation of LTM (Matsumoto et al., 2006). Taking these results into account, the LTM defect in aged crickets may accompany reduction in activation of the NO-cGMP pathway and cAMP pathway. To determine whether AMI of LTM in aged crickets can be rescued by applying activators of the NO-cGMP pathway or cAMP pathway, 3-week-old crickets in two groups were each injected with 3-μl saline containing 200-μM SNAP (NO donor), 200-μM 8br-cGMP (cGMP analog) or 200 μM 8br-cAMP (cAMP analog) into the hemolymph 20 min before two sets of differential conditioning. The estimated final concentrations of the drugs after diffusion were both 0.7 μM, calculated from injected volume and the approximate body weight of 850 mg. The timing of injection and the concentrations of the drugs were determined on the basis of our previous study (Matsumoto et al., 2006). As control groups, one group of 3-week-old crickets and another group of 1-week-old crickets were each injected with 3-μl saline alone prior to conditioning. The odor preferences of animals were tested before conditioning and 1 day after conditioning. Remarkably, all NO donor-injected aged crickets, 8br-cGMP-injected aged crickets and 8br-cAMP-injected aged crickets exhibited significant levels of retention at 1 day after conditioning (Figures 5Ac–e, *p* < 0.0001, WCX test), which were significantly greater than those in saline-injected aged crickets (compared to Figure 5Ab, *p* < 0.0001 in Figure 5Ac; *p* = 0.0002 in Figure 5Ad, *p* = 0.0007 in Figure 5Ae, M-W test) and were as high as that in saline-injected young adult crickets at 1 day after conditioning (Figure 5Aa, *p* = 0.7475 in Figure 5Ac; *p* = 0.4577 in Figure 5Ad, *p* = 0.4604 in Figure 5Ae, M-W test). The results suggest that an externally applied activator of the NO-cGMP pathway and cAMP pathway can rescue AMI in aged crickets. In contrast, recovery from AMI was not observed when these drugs were injected 20 min after training (SNAP, Figure 5Ba, *p* = 0.5525; 8br-cGMP, Figure 5Bb, *p* = 0.6791, WCX test), indicating that activation of the NO-cGMP pathway during or just after training is crucial for negating the effect of AMI.

### Aged Crickets can Retrieve Memory Formed Two Weeks Ago

**Figure 6:**
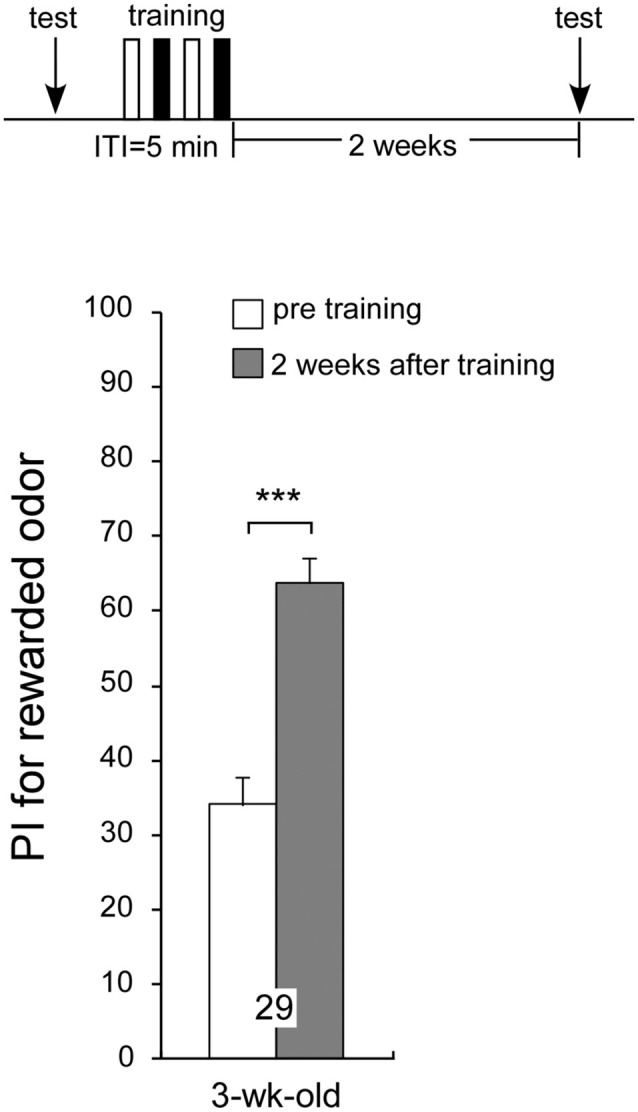
**Aged crickets can recall memory formed 2 weeks ago.** A 1-week-old cricket group was subjected to two sets of differential conditioning. The time schedule of the experiment is shown above the figure. PIs for rewarded odor before conditioning (white bar) and 2 weeks after conditioning (shaded bar) are shown as means ± SE. Statistical comparisons of odor preferences were made before and after conditioning (WCX test; ***p < 0.001).

Previous experiments have demonstrated that age-related impairment of LTM formation occurs in aged crickets; but does aging affect retrieval of LTM already formed in the past? To test this idea, 1-week-old crickets were subjected to two sets of differential conditioning and given a post-training odor preference test before conditioning and when they reached 3 weeks of age (2 weeks after conditioning) they exhibited a significant level of 2-week memory retention: preference for peppermint odor 2 weeks after conditioning was significantly greater than that before conditioning (Figure 6, *p* < 0.0001, WCX test), indicating that aged crickets can retrieve memory formed 2 weeks ago. Overall, the results suggest that aged crickets are able to retrieve memory consolidated in their early adulthood but that they fail to form LTM for novel association.

## Discussion

### AMI in Long-Term memory Formation

In this study, we showed that AMI occurs in crickets. Aged crickets failed to form LTM even when they received 4 sets of differential conditioning, which is twice the number of trials sufficient for young adults to form LTM (Matsumoto and Mizunami, 2002a; Matsumoto et al., 2003). This defect in aged crickets was observed in LTM formation but not in ARM formation. Aged crickets and young adult crickets did not significantly differ in total visiting time of odor sources, innate preference of vanilla over mint and in the ARM retention level. Therefore, motivation for odor exploration, locomotion ability, innate odor preference, odor segregation and learning ability did not differ between the aged and young adults. It is notable that once LTM is consolidated, aging will not affect its retrieval; aged crickets can normally retrieve memory formed in their early adulthood, but their memory about novel association decays within 4 h. This is the first demonstration in insects that aging does not impair the retrieval of consolidated LTM. The functional basis underlying LTM formation and retrieval may be independent, and aging may affect them differently. We conclude that crickets exhibit AMI of formation, but not maintenance or retrieval, of olfactory LTM.

In *Drosophila*, while ARM was not affected by aging, LTM exhibited AMI (Mery, 2007). This is consistent with our results in the present study. In addition, MTM is known to show AMI in *Drosophila* (Tamura et al., 2003). This MTM is one of the two memory components of ASM (another is the STM). A memory phase corresponding to this *Drosophila* MTM is yet to be clarified in crickets. ASM of crickets disappears within 20 min, making it difficult to investigate by the standard behavioral experiments that require 4 min for a testing session in the arena. However, we have recently developed a testing paradigm observing the maxillary-palpi extension response (MER; Matsumoto et al., 2015). It allows evaluation of memory acquisition and memory immediately after conditioning, similar to the PER paradigm in honey bees. With this MER paradigm, we are able to study if the memory within 20 min after conditioning is affected by aging.

### NO-cGMP Pathway in AMI

In the present study, we were able to fully restore the age-related decline in LTM level by injecting activators of the NO-cGMP pathway before training. Administration of these activators after training did not rescue AMI in LTM, indicating that the functional NO-cGMP pathway is required in LTM formation during or just after training. This injection time window-dependent effect of LTM induction by activators of the NO-cGMP pathway coincides with our previous results in young adult crickets. We have proposed that training crickets with multiple trials activates the NO-cGMP pathway, which activates the cAMP-PKA pathway leading to LTM formation via cyclic nucleotide-gated channels and Ca/calmodulin (CaM; Matsumoto et al., 2006). Therefore, we suggest that decline of NO production, which might be due to a reduction in activities of NOS or that of enzymes in upstream signaling pathways leads to a defect in LTM formation. However, whether the brain NO level actually falls in aged crickets is yet to be clarified. Our next challenge is to measure NO generation levels by electrochemical measurements using an NO-specific electrode and to measure NOS mRNA levels by applying real-time PCR to investigate whether the age-related decline actually occurs or not and, if it does, in which area of the brain it occurs.

Involvement of the NO-cGMP pathway in AMI suggested in this study has not been reported in any other invertebrates. In rats, it has been reported that activation of NO signaling restored memory impairment related to aging (Pitsikas et al., 2005). At the molecular level, many of the functions in memory formation processes are common between insects and vertebrates. For example, the AC-cAMP-PKA signaling pathway is thought to play an important role in memory formation in fruit-flies (Livingstone et al., 1984; Levin et al., 1992), crickets and honey bees (Müller, 2000; Matsumoto et al., 2014) as well as in rats. In mice, activities of AC1 (homolog of *rut* AC) and PKA expressed in the hippocampus decline with ageing (Angenstein et al., 1999; Karege et al., 2001a,b; Mons et al., 2004). Moreover, aged mice show a defect in hippocampus-dependent memory such as place memory; however the memory could be improved by cAMP activation in the hippocampus using a cAMP analog (Barad et al., 1998; Bach et al., 1999). Similarly, injecting a cAMP analog into aged crickets improved their AMI, suggesting a positive correlation between age-related degradation in functions of the cAMP signaling pathway and AMI. Controversially, there are several reports that suggest AMI improvement by inhibition of cAMP signaling such as age-related impairment of prefrontal cortex-dependent working memory in rats (Ramos et al., 2003) and olfactory MTM in fruit-flies (Yamazaki et al., 2007). Because vertebrates and insects share, at least in part, signaling pathways for LTM formation, crickets should serve as useful model animals for investigation of age-related LTM impairment. It is unknown whether AMI is due to degradation of NOS or molecules upstream of NOS activation. It is known that NOS is activated by activation of Ca/calmodulin. Despite the fact that Ca^2+^ influx in the brain is required for LTM formation in insects (Perisse et al., 2009), the type of receptor channel that regulates Ca^2+^ influx to activate NOS has not been specified. The best candidate now is the nicotinic acetylcholine (ACh) receptor from results of previous studies in insects (Bicker and Kreissl, 1994; Bicker, 1996; Goldberg et al., 1999; Zayas et al., 2002; Gauthier et al., 2006; Campusano et al., 2007; Dacher and Gauthier, 2008; Dupuis et al., 2012; Baz et al., 2013), and another candidate is *N*-methyl-*D*-aspartate (NMDA) subtype of the glutamate receptor as reported in rodents (Garthwaite, 1991; Bredt and Snyder, 1992; Snyder, 1992; Dawson and Snyder, 1994; Garthwaite and Boulton, 1995). ACh receptors (AChRs) can be subdivided into two subtypes: the metabotropic muscarinic AChR (mAChR) and ionotropic nicotinic AChR (nAChR). Honey bee nAChR are further classified into two subtypes according to their sensitivity to alpha-bungarotoxin (α-BGT): α-BGT sensitive nAChR and α-BGT insensitive nAChR. Pharmacological behavioral experiments by proboscis extension response revealed that α-BGT insensitive nAChR is involved in memory retrieval (Dacher et al., 2005; Gauthier et al., 2006). Similar results were also obtained in olfactory learning of cockroaches (Watanabe et al., 2011). On the other hand, α-BGT sensitive nAChR is suggested to be involved in LTM formation (Gauthier et al., 2006; Dacher and Gauthier, 2008). Honeybee α-BGT sensitive nAChR is also known to activate NOS via calcium ion (Bicker and Kreissl, 1994; Bicker, 1996; Dupuis et al., 2012). Our recent study by olfactory learning in crickets demonstrated that pharmacological inhibition of α-BGT sensitive nAChR did not affect ARM but inhibited LTM formation (Matsumoto, personal communication), as found in honey bees. Meanwhile, in honey bees, NMDA receptors (NMDAR) participate in formation of MTM and translation-dependent early-LTM, but not in late-LTM formation which is transcription- and translation-dependent (Si et al., 2004; Müssig et al., 2010). How NMDARs are involved in cricket olfactory learning is yet to be clarified. A search for LTM formation-related molecules that function upstream of the NO-cGMP pathway and testing whether the molecules display age-related changes are our next challenge.

### Brain Regions Involved in Insect AMI

Which regions in the cricket brain are responsible for AMI? Considering that AMI in crickets was restricted to LTM, it is reasonable to speculate that the region involved in LTM formation is the site where neural changes underlying AMI occur. The most likely brain area for LTM formation is the mushroom body, the secondary olfactory center, where one type of intrinsic neurons (Kenyon cells) exhibit a high level of expression of *NOS* mRNA, whereas other types exhibit a high level of expression of *sGCβ* mRNA (Takahashi et al., 2009). Another possible area is the antennal lobe, the primary olfactory center, in which a low level of *NOS* mRNA expression is found (Takahashi et al., 2009). It would be interesting to determine whether uncaging of NO in the mushroom body or the antennal lobe could rescue AMI.

### Trade-off Predicted Between Longevity and LTM Formation Ability

Half of the animals died within 2 weeks after the imaginal molt. Are animals with longer life span also more prone to exhibit AMI? In this case, being better at LTM formation would be at the expense of life duration. If this hypothesis is true, there is a possibility that NO plays a key role in this trade-off. According to the free radical theory of Harman, senescence is caused by cumulative oxidative stress by reactive oxygen species (ROS; Harman, 1956). NO reacts with superoxide, one of the ROSs, and generates peroxynitrite which promotes cell death and carcinogenesis (Lipton et al., 1993; Szabó and Ohshima, 1997; Afanasev, 2009). Thus, it is assumed that crickets with high NO level in the brain have a short life span; however, they maintain LTM formation ability. We therefore propose that a NO-mediated trade-off exists between LTM formation ability and longevity. A way to test this hypothesis would be to train 2-week-old adults and then correlate their LTM performance to their life duration and to perform quantitative analysis on brain NO levels.

### Cricket as a Model Animal of AMI Study

We have reported that crickets are insects with a high capability of olfactory learning and memory (Matsumoto and Mizunami, 2002b, 2004, 2005, 2006; Mizunami et al., 2009). Crickets have a relatively short life span after the imaginal molt (about 2 weeks on average; present work) among insect species that are used for studying learning and memory including *Drosophila* (6 to 7 weeks, Rogina et al., 2000; Tamura et al., 2003), cockroaches (about 30 weeks, Brown and Strausfeld, 2009) and honeybees (4 to 6 weeks in spring or summer workers, Fukuda and Sekiguchi, 1966; and 10 months in winter workers, Mattila et al., 2001). For studying AMI, the short life span of crickets has a great advantage. Further, a variety of learning paradigms with different conditioned stimulus (CS) and unconditioned stimulus (US) can be applied (Unoki et al., 2006; Nakatani et al., 2009) and may help to determine whether molecular processes underlying AMI in olfactory learning are general phenomena shared by other paradigms. To further investigate the nature of AMI, some aspects that crickets as a model animals, have are highly advantageous even among other insect species. Any conditions those alter cognitive functions other than the chronological age will be an obstacle for simple rendering of AMI features. Some insect species have physiological ages during adulthood, as seen in season-dependent maturation delay in migrating moths (Zhou et al., 2000) and occupation-dependent differences in cognitive functions of honeybees (Behrends et al., 2007; Behrends and Scheiner, 2010), both known to involve the juvenile hormone. Honeybees have drastic differences in adulthood life, such as social roles, season during which the bees emerged, gustatory responsiveness, and duration of foraging experience, and all these aspects have been shown to affect cognitive functions. For example, overwintering bees have life span much longer (>6 months) than the foragers in summer (<6 weeks), and exhibit slightly but significantly impaired olfactory LTM at 48 h after conditioning compared to that of the summer forager bees. Whether LTM impairment in this experiment is due to chronological age or physiological age is difficult to distinguish. In contrast, crickets are non-social insects having a relatively simple adulthood life, with less physiological differences among individuals especially after sexual maturation (completes at 3 days after the final molt (Sakai et al., 1990)). Therefore, we propose crickets as a pertinent model for studying neural mechanisms underlying AMI.

As crickets are omnivores and are hemimetabolic insects, learning novel odors in the late adulthood may not be their key priority. The AMI does not affect retrieval of memory gained during nymph stage (Matsumoto and Mizunami, 2002b) until the end of early adulthood (present study), and therefore the aged crickets would not have a problem searching for foods they already know. Male crickets are shown to exhibit mating behavior within 3 days after the imaginal molt, and become sexually competent (Sakai et al., 1990). The AMI, or in fact any event after mating, would be less sensitive to natural selection. This feature is common to many diseases including cancers, Alzheimer’s or Parkinson’s diseases which often occurs in humans after reproduction. As a result of the remarkable increase in life expectancy, post-reproduction period have elongated in humans. Crickets would be useful as a model animal to investigate the post-reproduction events less affected by natural selection, as they have relatively long and stable post-reproduction life.

## Author Contributions

YM designed and carried out experiments, analyzed and interpreted data, and wrote the manuscript. CSM carried out experiments, interpreted data and wrote the manuscript. TT carried out experiments, analyzed and interpreted data. MM designed experiments, interpreted data, and wrote the manuscript.

## Conflict of Interest Statement

The authors declare that the research was conducted in the absence of any commercial or financial relationships that could be construed as a potential conflict of interest.
